# Progress in Gynæcology

**Published:** 1903-04-18

**Authors:** 


### Progress in Gynæcology.

**Dysmenorrhœa.**—G. E. Herman and H. R. Andrews, in a paper on this subject, compared a number of cases of dysmenorrhœa cured by dilatation of the cervix with a number of others in which dilatation produced no benefit. The result of treatment they considered to be materially affected by the length of time the dysmenorrhœa had lasted, the age of the patient when treated, and the duration of her married life. In most cases cured by dilatation the time of commencement of the pain was almost synchronous with the commencement of the flow, while in most of the cases not cured the pain began two days or more before the flow. In half of those cured the pain had never lasted more than two days; in more than half of those not cured it had lasted more than four days. In four-fifths of those cured the pain was paroxysmal; in three-fourths of those not cured it was constant. They found no reason to think that dysmenorrhœa curable by dilatation was frequently associated with imperfect development of the uterus, or that there is such narrowing of the cervical canal as to hinder mechanically the flow of blood through it, but they thought it possible that some degree of smallness and rigidity of the canal might hinder its physiological dilatation during menstruation, and so provoke painful contraction of the uterine body.

**Repeated Ectopic Gestation.**—B. H. Wells[2] in an interesting paper on this subject reviews the literature, and arrives at the following conclusions:—Recurrent ectopic gestation is not extremely rare, and may be expected in a large proportion of pregnancies that occur subsequent to a tubal implantation of the ovum. That recurrence is liable to occur makes it necessary to watch with unusual care the early progress of any subsequent pregnancy. It is, he thinks, probable that the most frequent etiological factor leading to an abnormal implantation of the ovum is a mild inflammation of the tube, which, causing a change in its epithelial lining, increases the time occupied in the passage of the fertilised ovum through the tube.

**Intestinal Obstruction Caused by Transplanted Uterine Fibroid.**—Percy Paton[3] reports an interesting case in which a transplanted uterine fibroid gave rise to intestinal obstruction. The patient had the usual signs of intestinal obstruction. The abdomen was a good deal distended, and on the right side a tumour could be felt extending from the loin almost to the umbilicus. It was oval in shape, very hard, and could be moved from side to side; it did not seem to have any pelvic connection, although it felt very like a uterine fibroid. Several small fibroids could be felt in the uterus, but bimanual examination did not reveal any connection between the uterus and the tumour. The abdomen was opened and the tumour exposed; it was found to be attached to a considerable length of gut and mesentery, and as it was thought to be malignant and inoperable, the abdomen was closed. The patient died shortly after, and at the necropsy the tumour was found to be a transplanted fibroid of the uterus, from which organ it had become entirely separated. On the posterior surface of the uterus was to be seen a small stalk, and from this there could be little doubt the tumour had become disconnected. It had probably maintained its vitality through the extensive adhesion it had formed with the mesentery. There was a also considerable twist of the mesentery to which no doubt the constriction was to a very large extent due.

**Sterility.**—W. M. Polk[4] maintains that many cases of sterility are undoubtedly due to some abnormal condition of the uterine appendages. In many cases, as a result of past inflammation, gross structural changes have taken place in the tubes and ovaries; in others, however, the lesions are comparatively insignificant, there being nothing beyond some slight degree of distortion, fine adhesions, or slight stenosis of the abdominal ostium from adherent fimbriæ. When the more pronounced condition prevails symptoms are developed which, from the patient's point of view, take precedence of the sterility, while the lesser changes often give rise only to slight discomfort, accompanied however with sterility. Dr. Polk raises the question whether in these cases operation upon the appendages is justifiable with the object of curing the sterility. He thinks that when no other cause can be discovered to account for the sterility, it is quite justifiable to open the abdomen and deal with any abnormal condition of the appendages that may be present. He reports a number of successful cases operated on by others and one of his own. In his case he removed the right tube and ovary on account of disease, and freed the left tube from adhesions. Two years later the patient gave birth to a child. E. Gallant[5] recommends his uterine bi-valved drain for the treatment of sterility due to stenosis of the cervix. The instrument represents a split cylinder, the anterior and posterior sections being held together by means of a wire stem. The drain is $\frac{1}{4}$ inch in diameter, and $1\frac{3}{4}$ inch in length. It is introduced into the cervix with ordinary dressing forceps, and may be worn for several months, but it is well to remove, clean, and replace it after each menstrual period. Abram Brothers[6] writing on this subject also thinks that many cases of sterility are due to disease having extended from the uterus to the Fallopian tubes and peritoneal cavity, and in order to treat these successfully it is necessary to open the abdomen and deal with any diseased condition of the tubes and ovaries that may be found.

**Ovarian Pregnancy.**—Thompson[7] reports an interesting case of ovarian pregnancy. The patient was a II.-para, aged 32. She had not missed a period, but had suffered from severe pain in the left side of the abdomen for one month. On examination the uterus was found to be about its normal size, but there was a swelling on the left side, the nature of which was doubtful. An exploratory laparotomy was performed, when the left ovary was found to be enlarged, and on its inner and upper border there was a dark red tumour the size of a horse chesnut. The fimbriated end of the tube was free. The uterus was slightly enlarged. The ovary and tube were removed and the patient made a good recovery. Macroscopically the ovary was slightly enlarged, and the tumour was attached to its upper and inner end. Just inside the base of the tumour was found the follicle which had ruptured at the last period. The tube was patulous and its fimbriæ free. The fœtus, measuring 1·2 cm., was found attached by the umbilical cord to the wall of the ovisac. On section the ovary showed the unique combination of a pregnancy, a corpus luteum, and a recently-ruptured Graafian follicle. Several microscopical sections of the ovary, ovisac, and tube were made, and these helped to substantiate its claim to be an ovarian pregnancy. The tube showed no decidua, and was not concerned in the development of the pregnancy.

**Tuberculosis of the Genital Organs.**—Dr. Martin[8] maintains that tuberculosis of the female genitals is more frequent than is generally believed. It may occur primarily but is much more frequently secondary to tubercle elsewhere. Sometimes it enters by the vulva (ascending infection), but more often from the upper parts of the genital tract; it is very probable that infection frequently takes place through the intestine. There are no symptoms known to be pathognomonic of tubercle of the genitals, but when an inflammatory lesion exists there, in persons suffering from tuberculosis of other organs, there are grounds for suspicion that it too is tuberculous. As to prognosis this is always grave, but unless there is extensive destruction of tissue it is not hopeless. With regard to treatment, when the disease is extensive, and especially when other organs are affected, therapeutic measures should be limited to those of a general kind; when, however, the process is limited to the genitals, the extirpation of the diseased foci, or even the whole genital apparatus might be indicated, and should be carried out.

[1] Lancet, Dec. 27, 1902. [2] Med. Rec., Nov. 22, 1902. [3] Brit. Med. Jour., Jan. 17, 1903. [4] Med. Rec., Dec. 6, 1902. [5] Ibid., Nov. 22, 1902. [6] Post Graduate, June, 1902. [7] Med. Chron., Aug., 1902. [8] Lancet, Nov. 22, 1902.

